# Forastero cacao bean extract gel decreases IL-6 positive osteoblasts, osteoclasts, and osteocytes on OIRR model: A primitive study

**DOI:** 10.1016/j.jtumed.2025.02.005

**Published:** 2025-03-02

**Authors:** Shierin V. Fiolita, Firda Q. Aini Rasyid, Lilis Nurhalifah, Rudy Joelijanto, Leliana S. Devi, Vanda Ramadhani, Happy Harmono, Banun Kusumawardani, Syafika N. Fadiyah, Millenieo Martin

**Affiliations:** a Department of Dentistry, Faculty of Dentistry, University of Jember, Jember, Indonesia; b Department of Orthodontics, Faculty of Dentistry, University of Jember, Jember, Indonesia; c Department of Biomedical Sciences, Faculty of Dentistry, University of Jember, Jember, Indonesia

**Keywords:** Alveolar bone, Anti-inflammatory, Flavonoid, Forastero cacao bean (*Theobroma cacao* L.) extract gel, NF-κB, Orthodontic tooth movement, العظم السنخي, مضاد للالتهابات, الفلافونويد, ان اف-كابا بي, حركة الأسنان التقويمية, جل مستخلص حبة الكاكاو الفوراستيرو

## Abstract

**Background:**

Malocclusion can be corrected with orthodontic treatment to improve function and aesthetics. Orthodontic treatment patients have the potential to produce greater Reactive Oxygen Species (ROS), as evidenced by an increase in the Oxidative Status Index (OSI) a week after treatment. As a result, oxidative stress may induce an imbalance of bone remodeling through intensifying osteoclastogenesis by expressing pro-inflammatory cytokine, namely Interleukin-6 (IL-6). The worst pathological condition that can occur is Orthodontic-Induced Root Resorption (OIRR), a resorptive area on the root that is unwanted from orthodontic treatment. OIRR incidence is reported to be more than 90 % asymptomatic. Using Forastero cacao bean (*Theobroma cacao* L.) extract gel as a natural antioxidant and anti-inflammatory compound is expected to be able to prevent excess ROS, which triggers an increase in various pro-inflammatory cytokines, especially IL-6, which plays a role in the bone resorption pathway, thus reducing the incidence of OIRR and making orthodontic treatment successful through balanced bone remodeling.

**Aim:**

This study aimed to investigate the effect of Forastero cacao bean (*Theobroma cacao* L.) extract gel on the decrease of IL-6 cytokine expressed by osteoblasts, osteoclasts, and osteocytes in the OIRR model of male Wistar rats.

**Results:**

IL-6 positive osteoblasts, osteoclasts, and osteocytes decreased significantly after 8 % Forastero cacao bean extract gel-treated, as seen in T_7_ and T_14_ groups compared to C_-7_ and C_-14_ groups (p < 0.05).

**Conclusions:**

8 % Forastero cacao bean extract gel decreased IL-6-positive osteoblasts, osteoclasts, and osteocytes in the tension side of the alveolar bone.

## **الملخص**

**أهداف البحث:**

يمكن تصحيح سوء الإطباق بالعلاج التقويمي لتحسين الوظيفة والجماليات. لدى مرضى العلاج التقويمي القدرة على إنتاج أنواع الأكسجين التفاعلية بشكل أكبر، كما يتضح من زيادة مؤشر الحالة التأكسدية بعد أسبوع من العلاج. ونتيجة لذلك، قد يؤدي الإجهاد التأكسدي إلى اختلال التوازن في إعادة تشكيل العظام من خلال تكثيف تكوين الخلايا العظمية الناقضة للعظم من خلال التعبير عن السيتوكين المؤيد للالتهابات، وهو إنترلوكين-٦. أسوأ حالة مرضية يمكن أن تحدث هي امتصاص الجذر المستحث بواسطة تقويم الأسنان، وهي منطقة امتصاص على الجذر غير مرغوب فيها من العلاج التقويمي. تم الإبلاغ عن أن معدل حدوث امتصاص الجذر المستحث بواسطة تقويم الأسنان يزيد عن ۹۰٪ بدون أعراض. من المتوقع أن يكون استخدام جل مستخلص حبة الكاكاو فوراستيرو كمضاد للأكسدة الطبيعي ومركب مضاد للالتهابات قادرا على منع زيادة أنواع الأكسجين التفاعلية، مما يؤدي إلى زيادة في العديد من السيتوكينات المؤيدة للالتهابات، وخاصة إنترلوكين-٦، الذي يلعب دورا في مسار امتصاص العظام، وبالتالي تقليل حدوث امتصاص الجذر الناجم عن تقويم الأسنان وجعل العلاج التقويمي ناجحًا من خلال إعادة تشكيل العظام المتوازنة.

**طريقة البحث:**

هدفت هذه الدراسة إلى التحقيق في تأثير جل مستخلص حبة الكاكاو فوراستيرو على انخفاض سايتوكين إنترلوكين-٦ المعبر عنه بواسطة الخلايا العظمية والخلايا الناقضة للعظم والخلايا العظمية في الجانب المشدود من العظم السنخي في نموذج امتصاص الجذور الناجم عن تقويم الأسنان لفئران ويستار الذكور.

**النتائج:**

انخفضت الخلايا العظمية الموجبة للإنترلوكين-٦، والخلايا الناقضة للعظم، والخلايا العظمية بشكل ملحوظ بعد المعالجة بجل مستخلص حبة الكاكاو الفوراستيرو بنسبة ۸٪، كما هو موضح في المجموعتين تي٧ و تي۱٤ مقارنة بالمجموعتين سي٧ و سي۱٤.

**الاستنتاجات:**

أدى جل مستخلص حبة الكاكاو الفوراستيرو بنسبة ۸٪ إلى انخفاض الخلايا العظمية الموجبة للإنترلوكين-٦، والخلايا الناقضة للعظم، والخلايا العظمية في الجانب المشدود من العظم السنخي.

## Introduction

Malocclusion is a condition that deviates from the “ideal” occlusion. It refers to the abnormal alignment of the teeth resulting in disharmony between the maxillary and mandibular arches.^1^^,^^2^ According to the World Health Organization (WHO), the prevalence of malocclusion is estimated between 39 % and 93 % in children and adolescents. It is the third most prevalent oral health problem, following dental caries and periodontal disease.^3^ Malocclusion can be corrected with orthodontic treatment to improve function and aesthetics by moving the teeth to the “right position”. The most accepted theory of orthodontic tooth movement is the pressure-tension theory, which describes the alveolar tissue reaction induced by orthodontic forces that cause different responses in the bone. It can be divided into bone resorption on the compression side and bone deposition on the tension side of the alveolar bone. Bone remodeling occurs due to the resorption and deposition of alveolar bone, which is regulated by osteoblasts, osteoclasts, and osteocytes.^4^, ^5^, ^6^ After the orthodontic appliance is inserted, an inflammatory response occurs due to pressure. Inflammatory mediators that play a role in this response act as potential stimulators of ROS production in the oral cavity.^7^ According to a recent study by Primožic et al. (2021), orthodontic treatment patients have the potential to produce greater ROS, as evidenced by an increase in the OSI a week after treatment.^8^

When ROS is produced significantly above basal levels, the antioxidant defense capacity can trigger oxidative stress. As a result, oxidative stress may induce an imbalance of bone remodeling in orthodontic tooth movement through intensifying osteoclastogenesis by the expression of pro-inflammatory cytokine, namely IL-6, which will activate Receptor Activator of Nuclear Factor Kappa Beta Ligand (RANKL).^9^^,^^10^ The worst pathological condition that can occur is OIRR, a resorptive area on the root that is unwanted from orthodontic treatment. OIRR incidence is reported to be more than 90 % asymptomatic, which causes the shortening of the root to the weakening of the tooth structure. Even Dawood et al. (2023) stated that in some advanced cases, OIRR can ruin the affected tooth's long-term prognosis and impact the patient's quality of life.^11^

Therefore, it is essential to provide natural antioxidant compounds from outside the body derived from plants. Cacao (*Theobroma cacao* L.) is a tropical plant that the market has experienced massive growth in global supply over the last decade and its potential to be utilized.^12^ In Indonesia, cacao is one of the most important agricultural export commodities with massive growth.^13^ The most commonly cultivated cacao is Forastero, called Forastero cacao, which accounts for about 80 % of the cacao beans cultivated worldwide.^14^^,^^15^ In Indonesia and worldwide, cacao beans are exported raw; the rest is used as food, namely flour, butter, pasta, and cocoa foods. Cacao is also known as the primary source of the main ingredient for making chocolate. The nutritional content of cacao beans has been widely known and researched as an ingredient in herbal medicine for quite a long time. Cacao has even been dubbed a potential Nutraceutical (derived from the words “nutrition” and “pharmacy”) herbal plant. Nutraceuticals are getting much attention nowadays because of their potential for nutrition and therapeutic effects.^16^^,^^17^ Cacao beans contain many polyphenolic compounds, including flavonoids as their primary component; these compounds can be used as antioxidants, anti-inflammatory, antibacterial, antimutagenic, and anti-cariogenic.^18^^,^^19^ This content, especially as a good antioxidant and anti-inflammatory, has been proven, according to some studies, including the ability to suppress ROS formation and reduce pro-inflammatory cytokines such as Interleukin-6 (IL-6).^20^ Ahmed et al. (2020) also stated that the flavonoid content in cacao extract significantly reversed membrane peroxidation, nitro-oxidative stress, and decreased inflammatory markers (IL-6 and NF-kB).^21^

Various methods are used to prevent, minimize, or potentially repair OIRR using drugs and even lasers. Using cacao as a natural antioxidant and anti-inflammatory compound is expected to be able to prevent excess ROS, which triggers an increase in various pro-inflammatory cytokines, especially IL-6, which plays a role in the bone resorption pathway, thus reducing the incidence of OIRR and making orthodontic treatment successful through balanced bone remodelling. However, no studies have proven its function to show a decrease in IL-6 cytokine in osteoblasts, osteoclasts, and osteocytes under OIRR. This study aimed to investigate the effect of Forastero cacao bean (*Theobroma cacao* L.) extract gel on the decrease of IL-6 cytokine expressed by osteoblasts, osteoclasts, and osteocytes on OIRR model of male Wistar rats.

## Materials and Methods

This research was conducted following the approval from The Ethics Committee of the Medical Research Faculty of Dentistry University of Jember (No.1763/UN25.8/KEPK/DL/2022). Our experimental procedure, especially the rats-treated, has already followed our animal ethical protocol institution.

### Experimental animal groups

**Figure 1:**
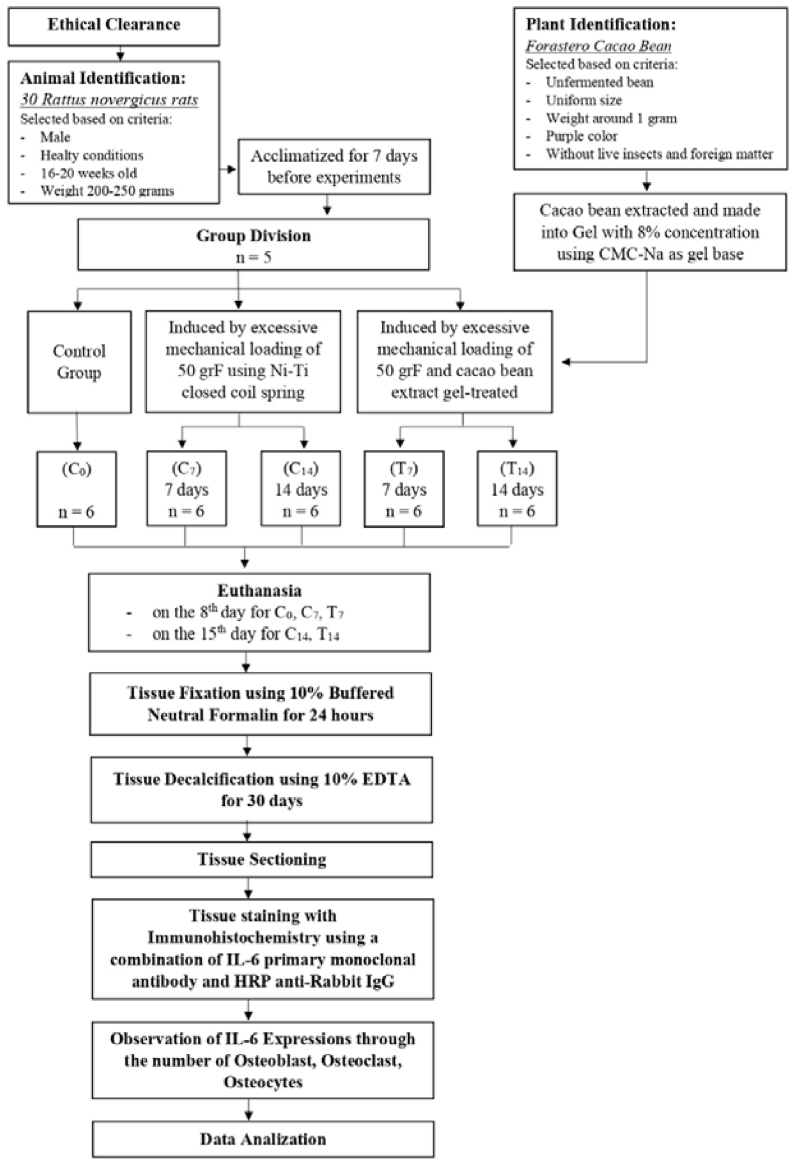
Study design scheme.

This study used the posttest-only control group design, as shown in Figure 1, with thirty male *Rattus norvegicus* rats (16–20 weeks old, 200–250 g) in healthy conditions and acclimatized for 7 days before starting the experiment. They were provided with an ad libitum diet and water.^22^ Rats were randomly divided into 5 groups, each consisting of 6 rats, with the following conditions: control group (C_0_), only orthodontic force was applied for 7 days (C_-7_) and 14 days (C_-14_), and treatment group with orthodontic force application and 8 % (80 mg/mL) Forastero cacao bean extract gel-treated for 7 days (T_7_) and 14 days (T_14_). Rats with unhealthy or dead conditions later were not included in the calculation.

### Forastero cacao bean extraction

Unfermented Forastero cacao beans that Jember State Polytechnic Integrated Agricultural Development has identified with reference number (No.227/PL17.8/PG/2022) are sorted based on Indonesian National Standard (SNI-01-2323-2008) so that good quality cacao beans are found with uniform size, without live insects and foreign matter, has a purple color, and has weight around 1 g.^23^ The selected Forastero cacao beans were dried and coarsely chopped, then dried again in an oven at 50 °C for 7 h to become even drier. The dried Forastero cacao beans are then ground in a blender until they become powder and sifted using a sieve to make the powder finer, called simplicia. This simplicia was then extracted by maceration method using 96 % ethanol (1:4 g/ml) for 3 days. The filtrate was evaporated using a rotary evaporator at 40 °C for 2 h to get a condensed extract.^24^^,^^25^

### 8 % (80 mg/mL) forastero cacao bean extract gel production

The condensed extract was made into a gel with a concentration of 8 % using CMC-Na as the gel base, which, according to research by Isnandar et al. (2022), can reduce the inflammatory process in bones and enhance osteoblasts. The Faculty of Pharmacy, University of Jember has certified the gel preparation and all the tests (No.03/B/Farm/X/2022). The entire gel-making process is carried out aseptically in Laminar Air Flow (LAF), starting with making the gel base by dissolving 3 g of CMC-Na powder in 100 ml of warm distilled water (70 °C), then leaving it to expand and stirring until homogeneous. To make 8 % cacao bean extract gel, add 4 g of cacao bean extract homogeneously to 46 g of prepared gel base.^26^

The results of cacao gel with a concentration of 8 % are then tested:

#### Organoleptic tests

Organoleptic tests are carried out by visually observing the preparation's odor, color, and texture. The results of the organoleptic test of the gel preparation are the criteria for a good gel preparation in terms of organoleptic aspects, namely a characteristic smell of cacao with a clear brown color and no syneresis.^27^

#### Homogeneity test

The homogeneity test is carried out by weighing 0.1 g of the gel preparation and then smearing it on a glass object or other object suitable for transparent materials. The composition is observed, and it is determined that the preparation shows a homogeneous sequence and no coarse grains are visible. The homogeneity results of the gel preparation met the criteria for a homogeneous gel preparation.^27^

#### pH test

The test was carried out using a universal pH indicator by immersing it in 1 g of gel sample, diluting it with distilled water to 10 ml, and making three replications. The universal pH will change color, and the pH measurement results will be obtained by adjusting the visible color to the universal pH standard. The pH value of the gel preparation was within the neutral pH limit (result 6.8). It shows that the gel is compatible with the oral mucosa and can be used without irritation.^28^^,^^29^

### Animal model establishment

**Figure 2:**
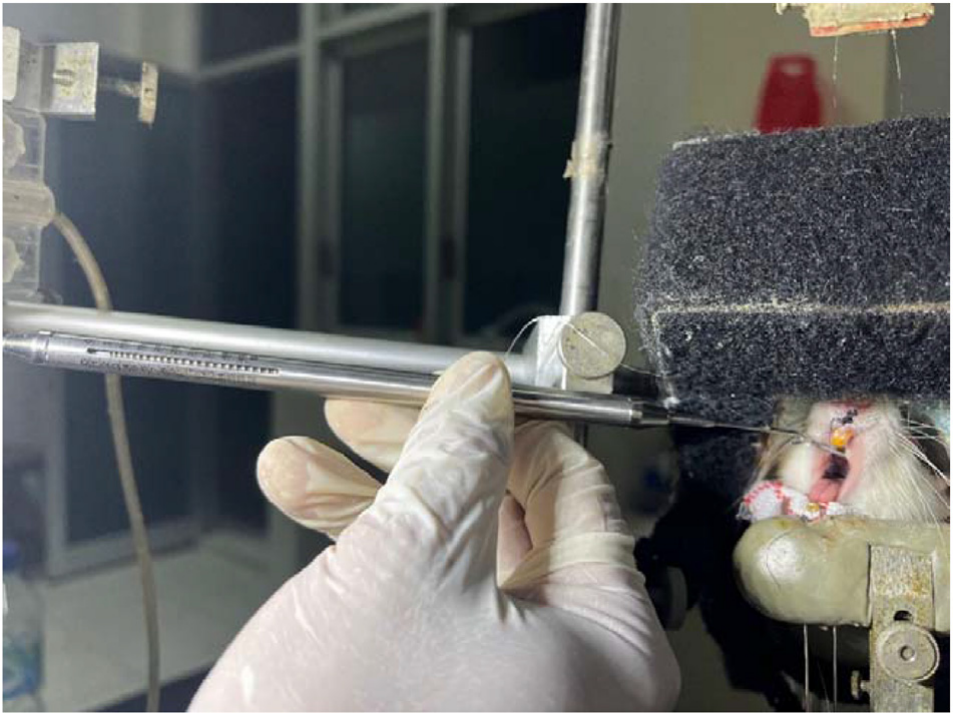
Excessive mechanical load of 50 grF as measured by a tension gauge.

**Figure 3:**
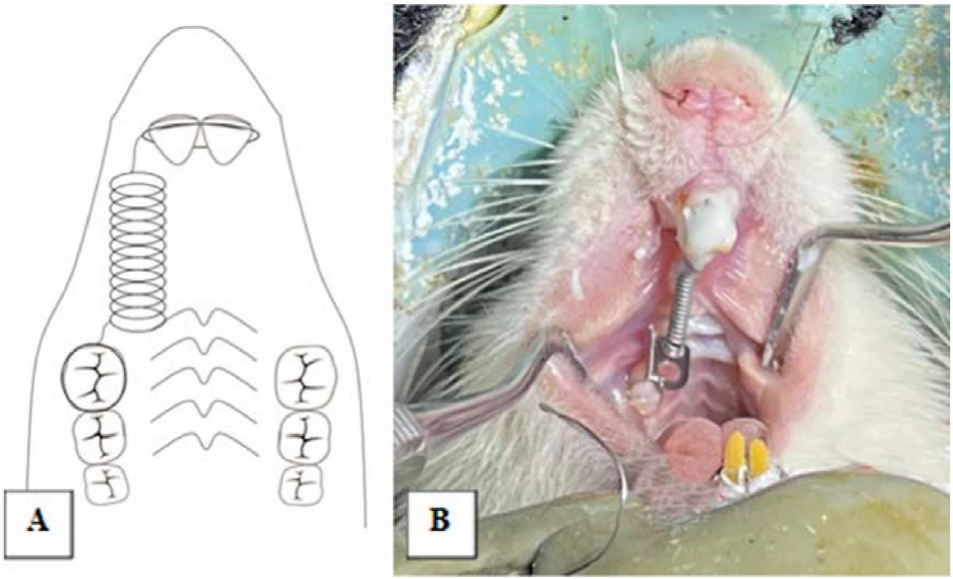
Animal model of orthodontic treatment. (A) The design of orthodontic appliance in rat. (B) The rat's intraoral photograph after the application of orthodontic appliance.

Induction of orthodontic forces in rats that had been anesthetized using 0.05 ml of a mixture of 10 % ketamine (25 mg/kg W) and 2 % xylazine (5 mg/kg W) was carried out by attaching a Nickel Titanium (NiTi) closed coil spring (6 mm; 0 .01 inch) between the maxillary incisor and the maxillary right first molar to move the maxillary incisor in the direction of the coil. Nickel Titanium (NiTi) closed coil spring was previously measured with a Tension Gauge (Ormco® Glendora, USA) to obtain an excessive mechanical loading of 50 grF^30^ (Figure 2). The device is fixed with stainless steel ligature wire with a diameter of 0.2 mm and bonded using glass ionomer cement to increase anchorage and prevent the device from dislodging. Control over the use of orthodontic appliances is carried out every day.^22^^,^^23^ The final result of installing an orthodontic appliance as a mechanical force on rat teeth is shown in Figure 3.

### 8 % (80 mg/mL) forastero cacao bean extract gel-treated

Forastero cacao bean extract gel was applied topically into the gingival sulcus of the maxillary incisors of male Wistar rats in the tension side using the blunted syringe needle until the sulcus was full twice a day (7 AM & 4 PM) for 7 days (T_7_) and 14 days (T_14_). The excess gel in the gingival sulcus was cleaned using a cotton pellet.^31^

### Tissue preparation

Animals were sacrificed after orthodontic treatment and extract gel administration on the 8th day for C_0_, C_-7_, and T_7_ groups and on the 15th day for C_-14_ and T_14_ groups chemically using a lethal dose of ketamine injection, about three times the anesthetic dose (120–150 mg/kg) intraperitoneally. Then, samples were dissected carefully using a blade and scalpel in the maxilla, including the incisor region of each rat, and fixed in 10 % Buffered Neutral Formalin (BNF) for 24 h. Then, the tissue samples were decalcified in 10 % EDTA for 30 days. After decalcification, the samples were dehydrated step using alcohol series, cleared by xylene, and embedded using paraffin wax.^22^ Afterward, it was manually sectioned as a ribbon with a 5 μm thickness in a labio-palatal direction using a microtome to present the teeth and alveolar bone tissue in the tension side.^32^ Then, the tissue sections are floated on a water bath to stretch. Tissue sections are then placed on premium coated object glass to be dried in a slide warmer at 45 °C for at least 12 h.^33^

### Immunohistochemistry evaluation

Tissue sections were routinely stained with immunohistochemistry using a combination of IL-6 primary monoclonal antibody and HRP anti-Rabbit IgG secondary antibody.^34^ Each stained tissue sample was first observed using a binocular microscope with 40 × and 100 × magnification to determine the observation area; under 400 × magnification, IL-6 positive expression within the tension side of the alveolar bone area was counted. The process was performed in five section areas, and the mean values were calculated. Then, the images were taken using an Optilab camera. IL-6 expressions were seen through the number of osteoblasts, osteoclasts, and osteocytes stained yellow-to-brown, a sign of IL-6 positive expression within the tension side of the alveolar bone area. The evaluation was assisted using the ImageJ application and calculated by three observers to avoid subjectivity.

### Statistical data analysis

The average number of IL-6 cytokines expressed by osteoblasts, osteoclasts, and osteocytes from the three observers were tested for normality using the Shapiro–Wilk test and homogeneity using the Levene test. As data were found to be normally distributed and homogeneous (p > 0.05), the parametric One-Way ANOVA test (p < 0.05) followed by the Least Significant Difference test was used to identify test groups that differed statistically significantly using SPSS software (26.0). The p-value <0.05 was considered statistically significant.

## Results

**Figure 4:**
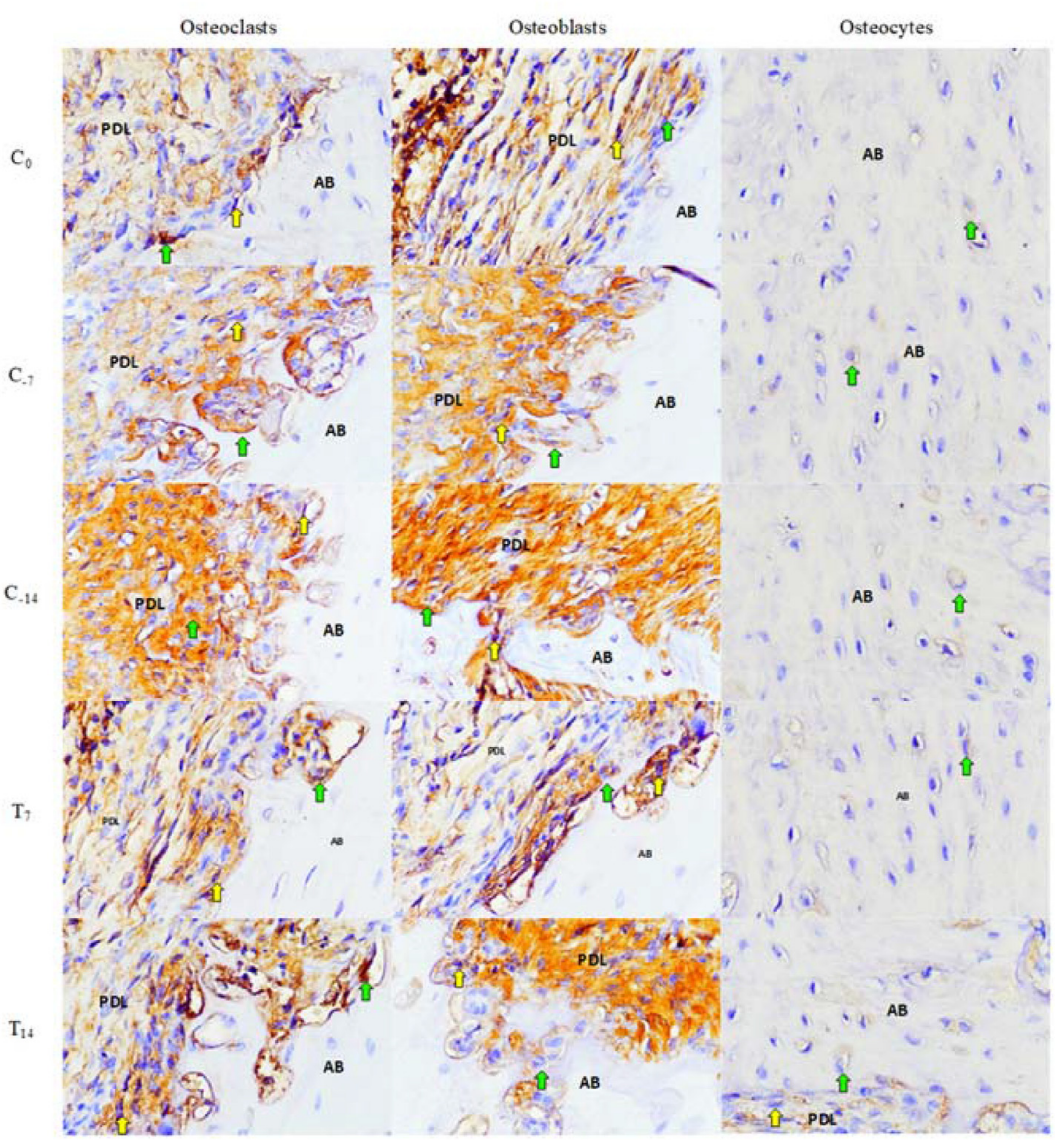
Immunohistochemistry observation for IL-6 expressed by osteoblasts, osteoclasts, and osteocytes using a light microscope at 400x magnification. Green arrows indicate IL-6 positive, and yellow arrows indicate IL-6 false positives. AB – alveolar bone, PDL – periodontal ligament.

The immunohistochemistry examination of IL-6, which was mainly observed within the tension side of the alveolar bone area of the rat's maxillary incisor, shows a positive reaction through the yellow-to-brown staining in osteoblast, osteoclast, and osteocyte in all experimental groups seen in Figure 4.

**Figure 5:**
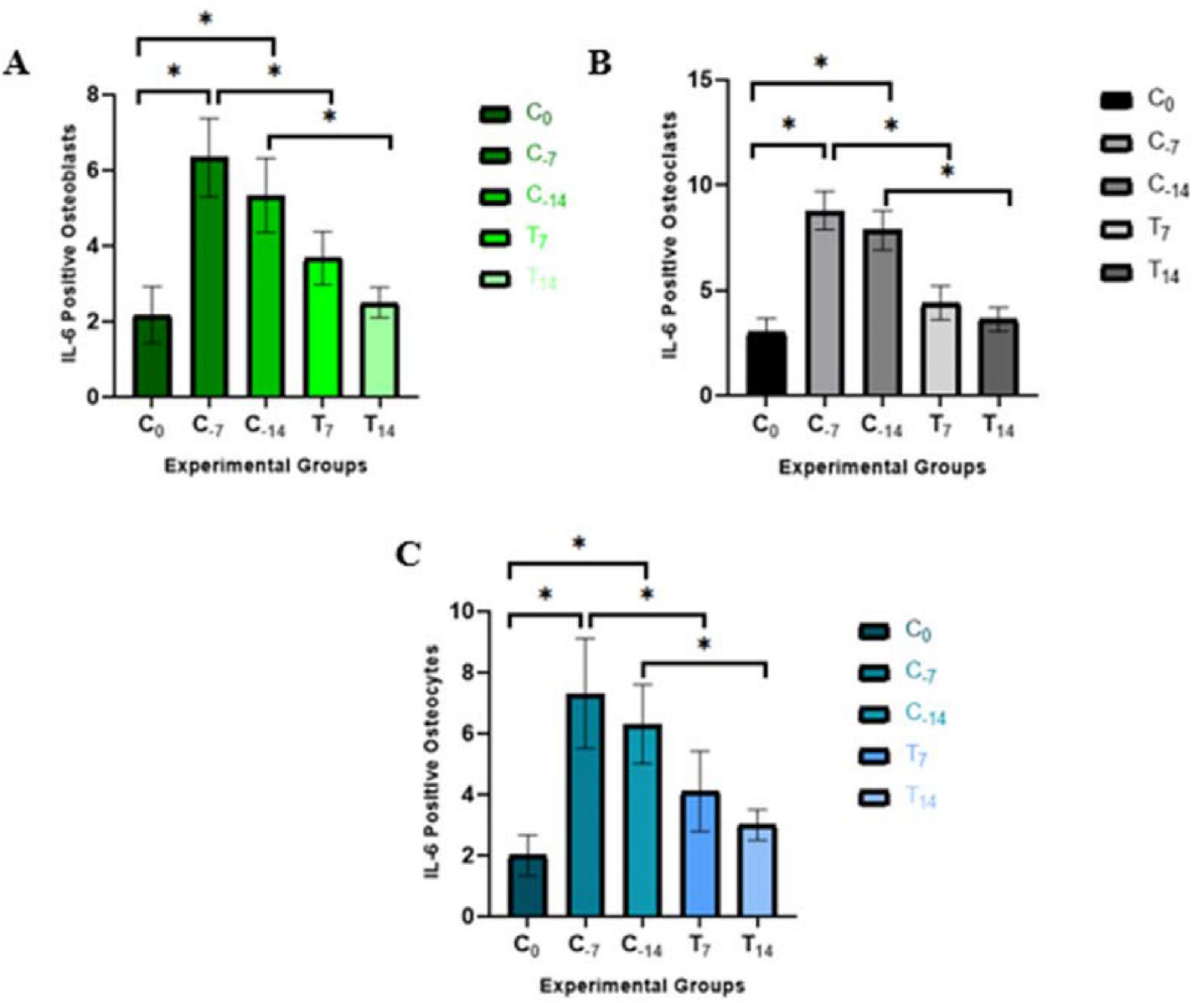
Bar chart of IL-6 positive (A) Osteoblasts, (B) Osteoclast, (C) Osteocytes between groups. Data are expressed as mean ± SD (∗P < 0.05).

Based on the calculated data, the average number of IL-6 cytokines expressed by osteoblasts, osteoclasts, and osteocytes in each group was obtained, as seen in Table 1. The mean of IL-6 cytokines expressed by osteoblasts, osteoclasts, and osteocytes increased significantly after applying orthodontic force on day 7 (C_-7_) rather than the control group (C_0_). It decreased on day 14 (C_-14_), as seen in Figure 5 and Table 2. Conversely, IL-6 positive osteoblasts, osteoclasts, and osteocytes decreased significantly after 8 % Forastero cacao bean extract gel-treated, as seen in T_7_ and T_14_ groups compared to C_-7_ and C_-14_ groups. Moreover, the T_14_ group showed fewer IL-6 positives than the T_7_ group, but the difference was insignificant. All the groups whose means are statistically different after the LSD test can be seen in Table 2. The longer duration of 8 % Forastero cacao bean extract gel-treated causes a lower amount of IL-6 cytokine expressed by the osteoblasts, osteoclasts, and osteocytes.

**Table 1:** Mean number and standard deviation of IL-6 expressed by osteoblasts, osteoclasts, and osteocytes between experimental groups.

IL-6 Positive	Groups				
	C_0_	C_-7_	C_-14_	T_7_	T_14_
Osteoblasts	2.17 ± 0.75	6.33 ± 1.03	5.33 ± 0.98	3.67 ± 0.70∗	2.50 ± 0.40∗
Osteoclasts	3.00 ± 0.67	8.78 ± 0.91	7.83 ± 0.94	4.39 ± 0.80∗	3.61 ± 0.57∗
Osteocytes	2.00 ± 0.67	7.30 ± 1.80	6.30 ± 1.30	4.10 ± 1.32∗	3.00 ± 0.50∗

C_0_: control group; C_-7_: only orthodontic force was applied for 7 days; C_-14_: only orthodontic force was applied for 14 days; T_7_: treatment group with orthodontic force application and 8 % (80 mg/mL) Forastero cacao bean extract gel-treated for 7 days; T_14_: treatment group with orthodontic force application and 8 % (80 mg/mL) Forastero cacao bean extract gel-treated for 14 days. ∗There is a significant difference (P < 0.05).

**Table 2:** LSD results between experimental groups.

Groups	IL-6 Positive Osteoblasts	IL-6 Positive Osteoclasts	IL-6 Positive Osteocytes
C_0_ – C_-7_	0.000∗	0.000∗	0.000∗
C_0_ – C_-14_	0.000∗	0.000∗	0.000∗
C_0_ - T_7_	0.017∗	0.003∗	0.010∗
C_0_ - T_14_	0.032∗	0.016∗	0.032∗
C-_7_ - C_-14_	0.102	0.037∗	0.187
C-_7_ - T_7_	0.009∗	0.000∗	0.004∗
C-_7_ - T_14_	0.004∗	0.000∗	0.001∗
C-_14_ - T_7_	0.000∗	0.000∗	0.000∗
C-_14_ -T_14_	0.000∗	0.000∗	0.000∗
T_7_ - T_14_	0.781	0.081	0.609

∗There is a significant difference (P < 0.05).

## Discussion

This study used the Rattus norvegicus Wistar strain as a minimal orthodontic treatment model, which is considered an adequate model with translational potential and widely used in the study of OTM and pharmacology.^1^^,^^35^ Male rats are used to avoid research bias due to the influence of the hormone estrogen. The animals were induced by 50grF orthodontic force, which is the amount of force that can move the teeth while producing OIRR conditions, according to research by Yu et al. (2019).^30^ The OIRR condition is caused by excessive resorption, characterized by increased expression of IL-6.^36^^,^^37^

Orthodontic tooth movement involves various cells in the dentoalveolar complex, especially cells that support and play a role in the resorption and apposition of alveolar bone (remodeling process), including osteoblasts, osteoclasts, and osteocytes through the release of cytokines, one of which is IL-6. IL-6 plays an important role, especially at the beginning of orthodontic tooth movement, and it is considered a classic pro-inflammatory cytokine that plays a role in bone resorption in the alveolar bone remodeling process and is a crucial mediator in the acute inflammatory response.^38^^,^^39^ IL-6 can activate osteoclast formation directly by binding to IL-6R on osteoclast and indirectly by stimulating the expression of RANKL by osteoblasts.^40^^,^^41^ RANKL is a crucial mediator of osteoclast differentiation and bone resorption by binding to RANK.^42^^,^^43^

Based on the research data, IL-6 positive osteoblasts, osteoclasts, and osteocytes in the control group (C_0_) showed the lowest number compared to the other groups because the rat model was in a condition without intervention. The number of IL-6 positive osteoblasts, osteoclasts, and osteocytes increased significantly (p < 0.05) in the group with the orthodontic mechanical force for 7 and 14 days (C_-7_ and C_-14_), as seen in Table 2 because immediately after applying the Ni–Ti closed coil spring on the rat's teeth, the initial phase of tooth movement occurs for 1-2nd days and lasts for 14 days. The pressure by the orthodontic appliance causes mechanical disturbances in the blood vessels so that necrotic tissue is formed and stimulates the release of various inflammatory mediators, one of which is the pro-inflammatory cytokine IL-6.^44^^,^^45^ In vivo, the study by Bletsa in Yamaguchi (2021) reported that IL-6 levels were increased in the periodontal ligament and alveolar bone after applying orthodontic mechanical force.^46^

In the T_7_ and T_14_ groups, an 8 % Forastero cacao bean extract gel was given. Gel form was chosen because of its easy application topically and faster absorption locally in the target tissue without going through the digestive system and being metabolized by the liver, which can increase the bioavailability of the extracted content. The gel will first be absorbed through the gingival mucosa, which consists of the lining of the oral epithelium, and then will continue to the lamina propria with tiny capillaries. These vessels carry drugs that are absorbed along with the blood through these capillaries to the alveolar bone as the intended target.^47^ Only a few studies have examined the efficacy of cacao bean extract gel, especially comparing various types of concentrations to find out whether a dose-dependent effect applies. Based on this, various concentrations of cacao bean extract gel are recommended for further research. The 8 % concentration in this study was chosen based on previous research by Isnandar et al. (2022), which found that it can reduce the inflammatory process in bones and increase osteoblasts.^48^ Apart from that, research by Kurniawati et al. (2019) showed that the administration of 8 % cacao bean extract gel was effective in reducing the inflammatory process, as evidenced by a decrease in the number of macrophages on tooth extraction wounds of male Wistar rats.^49^

This study showed a significant decrease (p < 0.05) in the amount of the cytokine IL-6 expressed by osteoblasts, osteoclasts, and osteocytes in the T_7_ and T_14_ groups compared to the C_-7_ and C_-14_ groups, as seen in Table 2. It means that the administration of 8 % (80 mg/mL) Forastero cacao bean (*Theobroma cacao* L.) extract gel is effective in reducing the number of IL-6 cytokines expressed by osteoblasts, osteoclasts, and osteocytes because of the polyphenol content, which includes flavonoids as its primary component. Flavonoids in Forastero cacao bean extract gel can act as both antioxidants and anti-inflammatories by reducing ROS and cytokines involved in inflammatory responses, which have been proven in line with various studies, both in vitro and in vivo.^50^ As an antioxidant, flavonoids in Forastero cacao bean extract were revealed by Zhang et al. (2022) have free radical scavenging activity by suppressing the formation of free radicals via inhibiting the enzymes involved in their formation, stabilizing the ROS that has been formed, thereby increasing defense protection through stimulation of antioxidant enzymes.^51^ These functions will then contribute to osteoblast differentiation and bone formation, maintain vital osteocytes that play a role in osteoblast activity and osteogenesis, and inhibit osteoclast activity and differentiation.^5^^,^^52^

**Figure 6:**
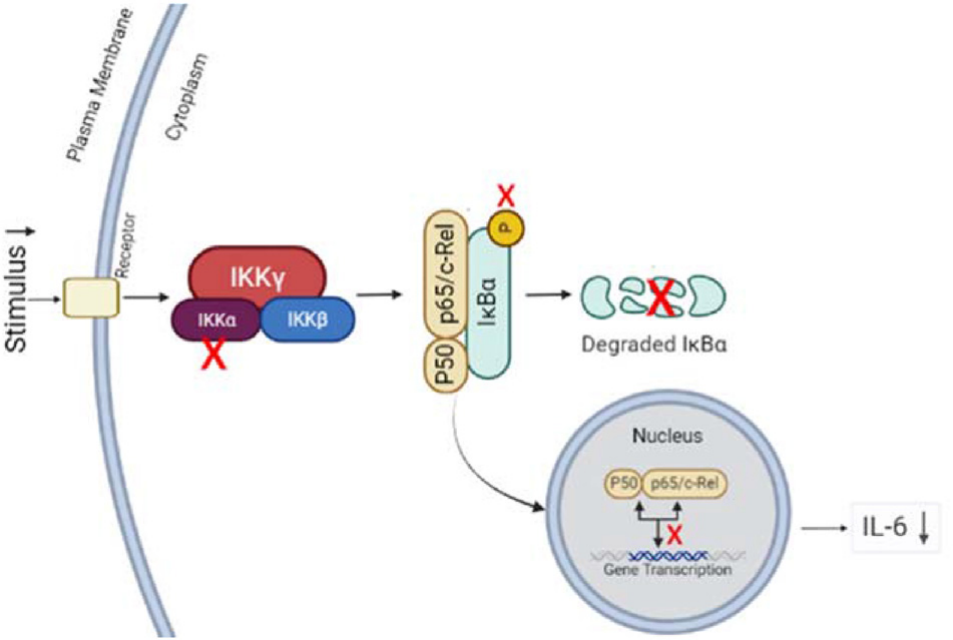
Mechanism of flavonoid in Forastero cacao bean extract gel as an anti-inflammatory via canonical NF-κB pathway.

When antioxidant mechanisms stabilize ROS, the role of Forastero cacao bean extract as an anti-inflammatory will also occur.^53^ As an anti-inflammatory, flavonoid content in cacao beans may reduce pro-inflammatory mediators, such as IL-6, by inhibiting the NF-κB pathway. NF-κB (Nuclear Factor Kappa Beta) is a transcription factor that plays a crucial role in modulating various biological processes, such as inflammation, through mediating the expression of cytokines and other inflammatory mediators.^54^^,^^55^ Direct control over NF-κB activity is exerted by the IκB kinase complex (IKK). The IKK complex consists of two catalytic components, IKKα and IKKβ, and one non-catalytic component, IKKγ. The mechanism for reducing NF-κB levels occurs when the content of cacao beans, namely flavonoids, reduces the inflammatory stimulus so that the IKK complex does not release one of its catalytic components, namely IKKα—the absence of IKKα impacts reducing the targeting of IκBα to phosphorylate. This decreased phosphorylation prevented IκBα from undergoing proteasomal degradation, followed by decreased activation of NF-κB to perform gene transcription in the nucleus.^56^^,^^57^ This decrease in activation will also reduce the expression of the resulting cytokines, including IL-6^54^ as seen in Figure 6.

Various pro-inflammatory cytokines produced as signals between cells in the immune system are interrelated during orthodontic tooth movement. These cytokines play an essential role in bone remodeling, which in this case is bone resorption, and in the occurrence of root resorption and exacerbation of OIRR. OIRR conditions involving multifactorial etiology still lead to excess pro-inflammatory cytokines as a manifestation. As mentioned earlier, the activation pathway for undesirable bone resorption will not occur when these inflammatory cytokines can be controlled.^58^ When the IL-6 cytokine expressed by osteoblasts, osteoclasts, and osteocytes decreases, there will be a decrease in RANKL production, which should bind to RANK on osteoclast precursors. This condition can inhibit osteoclast differentiation, proliferation, and excessive resorption, leading to OIRR. This reduction in excess osteoclastogenesis will create a balanced state of bone remodeling that is expected to increase the success of orthodontic treatment.^1^^,^^22^ Orthodontic treatment has the potential to produce an inflammatory process followed by oxidative stress, which then leads to OIRR, both due to the bracket system and the biomechanics of tooth movement. Administration of Forastero cacao bean extract gel has potential clinical applications through prooxidant-antioxidant homeostasis as a local pharmacological agent with both anti-inflammatory and antioxidant effects.^59^ Although there is only some research related to the cost-effectiveness of using cacao as a pharmacological agent, the fact that cacao is one of the largest commodities in the world makes it more reachable. The production of Forastero cacao bean extract gel in this study used the most straightforward procedures so that cost savings could be achieved. However, additional studies should include investigating Forastero cacao bean extract gel regarding its clinical use, scalability, and cost-effectiveness. The limitation of this study was that we only observed a decrease in the number of IL-6 as one of the pro-inflammatory cytokines that play a role in bone resorption and induce OIRR without directly measuring the percentage of resorption area. Besides, another molecular factor should be evaluated, considering that IL-6 was not the only factor in OIRR. Several types of tests should also be carried out to identify the overall composition of the cacao gel so that future research can highlight which ingredients work most significantly.

## Conclusions

Based on this study, we concluded that the administration of 8 % (80 mg/mL) Forastero cacao bean (*Theobroma cacao* L.) extract gel has a potential effect of preventing OIRR via the reduction of IL-6 positive, which is produced by osteoblasts, osteoclasts, and osteocytes in the tension side of the alveolar bone area of the rat's maxillary incisor during the application of orthodontic force. Moreover, it may also induce osteoblastogenesis, so this condition is expected to increase the success of orthodontic treatment.

## Ethical approval

All experimental animal procedures in this study were ethically approved by The Ethics Committee of the Medical Research Faculty of Dentistry University of Jember (No.1763/UN25.8/KEPK/DL/2022).

## Authors contributions

Shierin Velly Fiolita: Conceptualization, Data curation, Validation, Writing - original draft. Firda Qurrotul Aini Rasyid: Conceptualization, Data curation, Writing - original draft. Lilis Nurhalifah: Conceptualization, Data curation, Writing - review & editing. Rudy Joelijanto: Resources, Supervision, Validation. Leliana Sandra Devi: Investigation, Supervision, Validation. Vanda Ramadhani: Investigation, Supervision, Validation. Happy Harmono: Investigation, Resources, Validation. Banun Kusumawardani: Investigation, Resources, Validation. Syafika Nuring Fadiyah: Conceptualization, Data curation, Investigation. Millenieo Martin: Conceptualization, Investigation, Validation, Writing - original draft, Writing - review & editing. All authors have critically reviewed and approved the final draft and are responsible for the content and similarity index of the manuscript.

## Source of funding

This work was supported by the DIPA University of Jember [grant numbers 3213/UN25.3.1/LT/2023, 2023].

## Conflict of interest

All the authors hereby declare that there is no conflict of interest.
